# Longer sequence insertions in p6 *gag* play a role in immune escape in HIV-1 subtype C

**DOI:** 10.1186/1471-2334-14-S3-E7

**Published:** 2014-05-27

**Authors:** Shilpee Sharma, GA Shambhu Prasad, Ravi Vijaya Satya, Viswanath Ragupathy, Shanmugam Saravanan, Kailapuri G Murugavel, Pachamuthu Balakrishnan, Suniti Solomon, Indira Hewlett, Udaykumar Ranga

**Affiliations:** 1 Jawaharlal Nehru Centre for Advanced Scientific Research, HIV- AIDS Laboratory, Bengaluru, India; 2 Bioinformatics – Databases and Algorithm Development, Qiagen, MD, USA; 3 Food and Drug Administration, Laboratory of Molecular Virology, Bethesda, MD 20892, USA; 4 YRG Centre for AIDS Research and Education, Chennai, India

## Background

HIV-1 is capable of evading the CTL immune response by introducing mutations in residues both within the epitopes and in sequences flanking the epitopes. The present study aims at identifying the mechanisms of CTL immune escape primarily in the asymptomatic phase of HIV-1 subtype C in drug naive patients from an Indian clinical cohort.

## Methods

In a prospective study, a cohort of select seropositive drug naive subjects is being monitored at YRG CARE, Chennai for a period of two years with repeated sampling at 6-month intervals. The viral RNA was extracted from plasma and Gag was amplified, followed by Sanger sequencing. The samples of interest were further subjected to next-generation sequencing using Illumina MiSeq and analyzed using the CLC Genomics Workbench software.

## Results

Twenty plasmid clones of gag were sequenced from one of the subjects at four different time-points. We observed multiple viral strains that did or did not contain an insertion of 14 amino acid residues in the PTAP domain of p6 *gag*. The PTAP duplication was further confirmed in 6 other subjects using the next- generation sequencing.

## Conclusion

The preliminary data suggest that subtype C is capable of causing sequence insertions of longer length in the PTAP domain of the p6 *gag*, unlike other viral subtypes that insert sequences of shorter length at this location. The bio informatics analysis is suggestive of the role of the amino acid insertions in immune escape in the chronic phase of the HIV-1 infection.

